# Introducing a User-Friendly Technique for Treatment of Palatally-Impacted Canines with the Aid of Temporary Anchorage Devices

**DOI:** 10.30476/DENTJODS.2022.91156.1557

**Published:** 2022-12

**Authors:** Mohammad Noroozian, Mohsen Merati, Farzin Heravi, Hooman Shafaee

**Affiliations:** 1 Dept. of Orthodontics, Student Research Committee, School of Dentistry, Ilam University of Medical Sciences, Ilam, Iran; 2 Dept. of Orthodontics, School of Dentistry, Tehran Shahed University of Medical Sciences, Tehran, Iran; 3 Dept. of Orthodontics, School of Dentistry, Mashhad University of Medical Sciences, Mashhad, Iran; 4 Dept. of Orthodontics, Dental Research Center, School of Dentistry, Mashhad University of Medical Sciences, Mashhad, Iran

**Keywords:** Impacted teeth, Orthodontic anchorage, Canine tooth

## Abstract

Treatment of impacted canine teeth is a challenge for most clinicians, especially in adult patients with high risk of ankylosis. Conventionally, leveling and alignment of the teeth are first performed and after heavy arch wire placement in slot of brackets, de-impaction force is applied. However, this method has some disadvantages, such as inability to detect ankylosis of the impacted tooth until load application, risk of root resorption of incisors or necrosis of them, distortion of dental arch form, the need for early extraction of primary canines, which is esthetically unfavorable for patients, and long-term presence of fixed orthodontic appliances in the oral cavity. This study aims to introduce approach that can be easily used by busy clinicians to guide palatally impacted canines into the dental arch using a cantilever spring supported by two palatal miniscrews prior to the initiation of fixed orthodontic treatment, and report some cases treated with this technique. This technique does not have the shortcomings of the conventional technique. In addition, the total duration of treatment, and duration of presence of orthodontic appliances in the oral cavity are shorter than the conventional technique. In addition, is less technique sensitive and do not need time complex and time consuming wire bending

## Introduction

The maxillary canine teeth are the most commonly impacted teeth after the maxillary third molars. According to the literature, the prevalence of canine impaction varies from 0.92% to 1.7% [ 1 ]. It is slightly more common in females than males, and is more frequently seen unilaterally. The prevalence of palatal impaction of canine teeth is 2-3 times the rate of their buccal impaction [ 1 - 3 ]. Canine impaction has a multi-factorial etiology; environmental factors such as presence of a barrier or a pathological lesion against the path of insertion of canine as well as genetic factors play a role in its occurrence [ 4 - 5 ]. 

Several methods have been suggested for treatment of tooth impaction such as tooth extraction and its replacement with dental implant, surgical repositioning, and surgical-orthodontic approach [ 2 , 6 - 8 ]. 

Achieving a beautiful smile is among the most important reasons for patients seeking orthodontic treatment. The influential role of maxillary canine teeth in achieving a beautiful smile has been previously emphasized [ 9 - 12 ]. Accordingly, the forced eruption of canine is the ideal treatment option for canine impaction and is often preferred to other available treatment options [ 13 ]. 

Cone-beam computed tomography (CBCT) is highly efficient for detection of the correct position of the impacted canine, determining the most appropriate site of surgical exposure, and the proper direction of application of orthodontic forces [ 14 - 16 ]. Assessment of the position of the palatally impacted canine teeth relative to the roots of the adjacent central and lateral incisors, and first premolar, and presence of root dilaceration in the impacted tooth or the adjacent teeth is imperative prior to the initiation of orthodontic treatment. Root resorption of the lateral incisor adjacent to an impacted canine has been reported in 80% of the cases [ 17 ]. 

In the conventional forced eruption approach, the teeth are first leveled and aligned in order to be able to pass a heavy archwire through the bracket slots, and then the required force for de-impaction of the impacted canine is applied with the support of a heavy archwire. The applied force is distributed equally in the entire dental arch; thus, the side effects due to the reaction force, especially in teeth adjacent to the impacted tooth, decrease [ 18 ]. However, the conventional method has some drawbacks: bonding and engagement of the lateral incisor in orthodontic archwires at the onset of treatment may cause displacement and approximation of the tooth apex to dental follicle of the canine tooth. This results in contact of the root of the lateral incisor with the crown of the impacted canine tooth, which can initiate or aggravate root resorption. Thus, distalization and distancing of the impacted canine from the adjacent roots prior to the onset of fixed orthodontic treatment decreases the risk of further root resorption of the lateral incisor [ 19 - 20 ]. On the other hand, use of cantilever springs connected to transpalatal arch for de-impaction of impacted canine may lead to anchorage loss and mesialization of molars. If non-extraction orthodontic treatment is considered, it can lead to space shortage and prevent bringing the impacted canine into the dental arch [ 21 ]. In the conventional method, orthodontic brackets and wires are placed in the oral cavity since the onset of treatment. Thus, braces are present in the oral cavity for a relatively long time. This increases the rate of treatment complications such as decalcifications and root resorption [ 22 - 23 ]. Presence of braces in the oral cavity for the shortest time possible, and their invisibility would be a great advantage for adult patients [ 24 ]. On the other hand, ankylosis of an impacted canine tooth cannot be definitely determined based on radiographs. In case of ankylosis, the dental arch form is often distorted upon the application of de-impaction force. Correction of this condition is considered a type of round tripping and increases the duration of treatment. If dental impaction is the only problem requiring orthodontic treatment, an unnecessary course of fixed orthodontic treatment is also imposed on patient prior to extraction of impacted ankylosed tooth and its replacement with dental implant [ 25 - 26 ]. 

This study introduces a novel approach for treatment of palatally impacted maxillary canines without the disadvantages of the conventional method. This novel approach is composed of two phases namely a de-impaction phase and a comprehensive fixed orthodontic treatment phase.

## Case Presentation

### De-impaction phase

**Figure 1:**
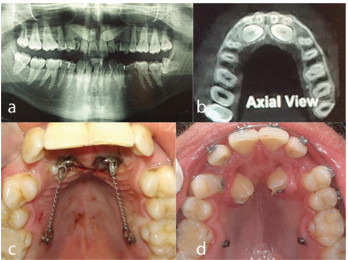
**a:** The Panoramic view, **b:** CBCT view. Note the proximity of the impacted canine to the incisor root; any tooth flaring following bonding may result in incisor root resorption, **c:** Impacted canine was surgically exposed, and force was applied immediately, **d:** A few months later, impacted canine ankylosis was ruled out and the upper arch was bonded

In the de-impaction phase, the impacted canine tooth is surgically exposed and bonded. Force applied from the mini-screw in the palate, distalizing, and guiding it toward the dental arch, simultaneously. Figure 1 shows the treatment steps in the de-impaction phase. After assessing the location and position of the impacted canine using panoramic radiography and cone-beam computed tomography, the relationship of the impacted tooth crown with the adjacent tooth root is determined, and the path of movement of the impacted tooth towards the dental arch is determined. Next, the impacted tooth is surgically exposed and the attachment is bonded to it. Comprehensive orthodontic treatment is not commenced until the impacted canine cusp tip emerges through the mucosa, because in case of early initiation of fixed orthodontic treatment and bonding of maxillary teeth, flaring of incisors in the process of leveling and aligning can cause incisor root resorption, considering the proximity of the impacted tooth crown and the incisor root (Figures 1a and Figure 1b). 

In the surgical treatment session, bone tissue should be removed to the height of contour of the tooth. Accordingly, the widest part of the tooth crown can be moved following load application with no barrier against it [ 27 ]. Presence of an orthodontist is recommended during the surgical procedure for the intraoperative bonding process. Considering the need for the tipping movement in distalization of canine crown and distancing it from the roots of the adjacent lateral and central incisors, the crown tip was considered as the most appropriate point for the attachment bonding. After exposure of the impacted canine and control of bleeding, the bonding area is first slightly roughened by a diamond bur to eliminate the prismless enamel and enhance enamel etching [ 28 ]. A button or bracket is bonded to the crown tip. A button-type miniscrew measuring 1.6×8 mm is placed between the roots in the palatal side perpendicular to the palate, by taking into account the location of the greater palatine nerve and vessels. Load application from the mini-screw to the impacted tooth is initiated immediately after the termination of surgical procedure using a NiTi closed coil spring or an elastic chain (Figure 1c). According to the authors’ experience, when ankylosis is suspected, an elastic chain can be used for load application since an elastic chain applies a heavier load than a NiTi closed coil spring. 

The impacted tooth is then distalized by load application from the mini-screw, and is distanced from the lateral incisor root. This step requires very little chair-time, and can be easily performed in a busy orthodontic office as full bonding of dental arch or any spring fabrication not required. This step has some other advantages. For instance, it enables the application of orthodontic force immediately after surgery and luxation of impacted teeth with small areas of ankylosis, which is imperative for prevention of reankylosis. Following application of a continuous and controlled force at first to the impacted tooth in this phase, we will soon see its movement and its emergence through the oral mucosa, which is very pleasant for patients and increases their motivation to continue treatment.

**Figure 2:**
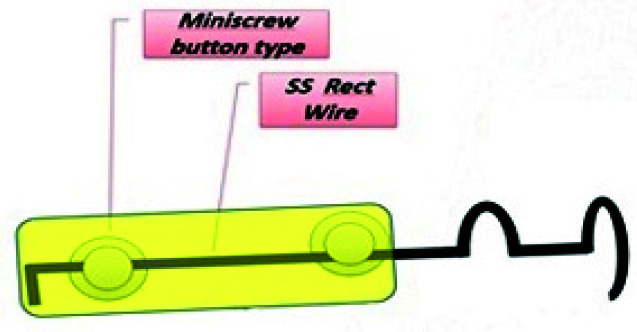
Cantilever spring design and its position relative to the miniscrews. Miniscrews, wire segment between them and distal end bend were covered with flowable light-cure composite resin to enhance patient comfort and increase the stability of the appliance

**Figure 3:**
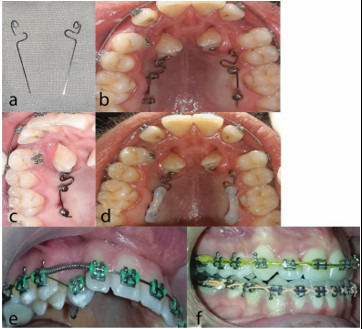
a: Cantilever spring design during a separate session with adequate chair-time, b: Cantilever spring was positioned. c: Canti-lever spring end was cinched, d: Spring was covered with flowable light-cure composite resin, e: Canine was moved into the prepared space with NiTi overlay wire, f: Finishing and canine torque correction by placing full-size rectangular wire

Monthly visits are then scheduled for the patient and after displacement of the impacted tooth and ensuring 

the absence of tooth ankylosis (Figure 1d), the clinician can place the second miniscrew, with properties similar to the first one, and fabricate a cantilever spring in a separate session with adequate chair-time. The cantilever spring is fabricated using 0.017×0.025-inch stainless steel wire, with a U loop in the middle to adjust the direction of load and a hook or helix in the anterior region to ligate the ligature wire. The spring passes through the occlusal part of the mesial miniscrew and enters the hole in the hex of the distal miniscrew (Figure 2). After adjusting the spring position and passing it through the distal miniscrew hole, the spring end was cinched to prevent its rotation, and stabilize its position (Figure 3a - Figure 3c). To prevent spring displacement, provide a stronger anchorage, and ensure patient comfort, flowable composite was uniformly applied over the two miniscrews, the wire segment between them, and the distal bend (Figure 3d). The advantage of placing the spring in the occlusal part of the mesial mini-screw is the approximation of the spring to the anterior miniscrew head during the activation of it. This eliminates the risk of composite fracture or wire deformation in the process of movement of impacted tooth towards the dental arch. By use of this spring, load can be applied in any direction and tooth can be moved to the distal, mesial, buccal, or lingual side, easily. Even if bracket is bonded, the impacted canine root torque can be corrected by engaging the wire into the bracket slot. During the next visits, the impacted tooth gradually guided towards the dental arch and its rotation corrected by the cantilever spring. 

According to the clinical experience of the authors, the appropriate site for placement of the first miniscrew is the palatal region between second premolar and first molar, because the surgical flap and postoperative inflammation often affect the palatal gingiva of first premolar. The palatal space between first molar and second molar is more appropriate for placement of the second miniscrew. The first or the mesial miniscrew may experience mesial migration and tipping following the application of high load required for de-impaction of impacted canine tooth. If so, passing the cantilever spring through the hex of this miniscrew would be difficult, and the contact of spring with it in the occlusal surface would suffice. 

### Comprehensive orthodontic phase

In patients with severe crowding of other teeth and other maxillofacial anomalies, it is recommended to commence comprehensive orthodontic treatment as soon as the impacted canine crown tip emerges through the mucosa. While the anchorage system supported by the palatal mini-screws guides the canine tooth towards the dental arch, leveling and aligning of other teeth are simultaneously accomplished. By doing so, the course of treatment is significantly shortened. Finally, the impacted canine reaches its final position in dental arch by the combined action of the cantilever spring in the palate and comprehensive orthodontic treatment. The buccal movement of the canine tooth is accomplished by the help of the heavy archwire base and ballista spring or NiTi overlay wire (Figure 3e). Finally, the canine root torque is corrected by placement of a rectangular wire in bracket slots (Figure 3f). 

Herein, we discuss several patients treated with this approach. In order to focus on the treatment technique employed for de-impaction of palatally impacted canine teeth, the pre- and postoperative patient documents and details of their malocclusion type are not discussed here.

### Patient 1

**Figure 4:**
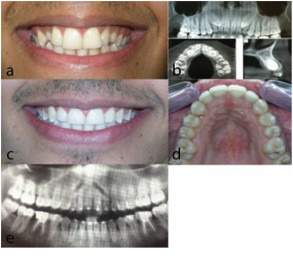
Patient 1. a: Frontal smile photograph before treat-ment. b: Panoramic view and cone beam computed tomogra-phy (CBCT) views. c: Frontal smile photograph after treat-ment, d: Occlusal photograph after treatment, e: Post treat-ment panoramic view (total treatment time: 23 months)

Our first case was a 24-year-old male with bilateral impaction of maxillary canine teeth. Figure 4 shows the presence of primary canine teeth and close proximity of the impacted permanent canines with the root of the lateral incisors. The patient had class I skeletal relationship, mild maxillary and mandibular crowding, and 3mm of midline deviation in the mandible relative to the maxillary skeletal and dental midline. The patient’s treatment plan included de-impaction of impacted maxillary canines, correction of crowding, and correction of midline deviation as much as possible without tooth extraction. The left canine had a 90° rotation, while the right canine had approximately 45° rotation relative to the dental arch. 

In the first treatment session, the canine tooth was surgically exposed and bonded. The palatal flap was sutured, and a window was created in the palatal mucosa to access the impacted canine. One button-type minis-crew was placed in each side between second premolar and first molar to apply distalizing force to the two palatally impacted teeth. Load application was initiated in the surgical session using an elastic chain with an approximate force of 250 g at each side. One month later, the canine cusp tip was visible through the palatal mucosa. At 3 months, the canine tooth was completely out of the mucosa. At this time, the second miniscrew was placed between the roots of first molar and second molar, and load application was continued by the cantilever supported by the mini-screws. Simultaneous with the movement of canines towards the dental arch, tooth rotation was initiated by controlling the direction of loads. Comprehensive orthodontic treatment was commenced at 7 months. At 12 months, the canine tooth was close to the dental arch. At this time, an open coil was used to create the required space for the permanent canine teeth in the dental arch using 0.020-inch stainless steel wire. During the entire 12-month treatment period, the primary canines were preserved since the patient was concerned about his smile esthetics. At 12 months, brackets were bonded to the buccal surface of both permanent canine teeth, and the primary canines were extracted. The canine teeth were guided into the dental arch using 0.014-inch and 0.016-inch NiTi overlay wires, respectively. The treatment was accomplished at 23 months (Figure 4). 

### Patient 2

**Figure 5:**
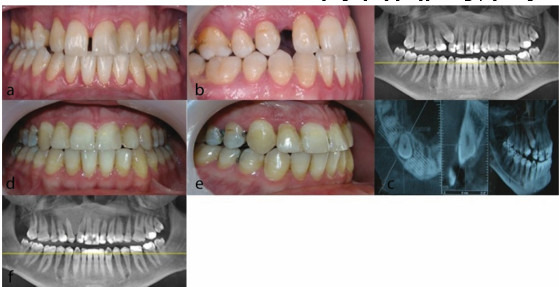
Patient 2. a: Frontal occlusion photograph before treatment, b: Lateral occlusion photograph before treatment, c: Panoramic and cone beam computed tomography (CBCT) views, d: Frontal occlusion photograph after treatment, e: Lateral occlusion photograph after treatment, f: Post treatment panoramic (total treatment time: 18 months)

Our second case was a 26-year-old male that presented with maxillary right canine impaction. He had mild class III skeletal relationship and class I dental malocclusion. The treatment plan included de-impaction of maxillary right canine and non-extraction correction of malocclusion. As shown in Figure 5, the impacted canine had a close contact with the lateral incisor root. Since the cingulum of the impacted tooth was sticking out of the palatal cortex, it was decided to guide the tooth from the palatal towards the buccal to preserve the buccal cortex. All procedures were performed according to the protocol. The site of placement of the first mini-screw and the method of load application were similar to our first case. After 1 month, the canine cusp tip emerged from the buccal mucosa. At 3 months, the canine tooth was completely out of the mucosa. At this time, the second mini-screw was placed between the roots of first molar and second molar, and the canine tooth was guided towards the arch with the help of a cantilever spring supported by two mini-screws. Comprehensive orthodontic treatment was started at 5 months. At 10 months, the canine tooth was close to the dental arch. Thus, the required space for the canine tooth was created in the dental arch using 0.020-ich stainless steel wire and an open coil. At 10 months, a bracket was bonded to the buccal surface of the canine tooth; it was guided into the dental arch using 0.014-inch, and then 0.016-inch NiTi overlay wires. The treatment was accomplished at 18 months (Figure 5).

### Patient 3

**Figure 6:**
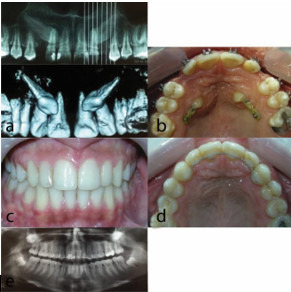
Patient 3. a: Panoramic and cone beam computed tomography (CBCT) views, b: A few months after and start-ing fixed orthodontic treatment, c: Frontal occlusion photo-graph after treatment, d: Lateral occlusion photograph after treatment, e: Post treatment panoramic (total treatment time: 19 months)

Our third case was a 13-year-old female with bilateral impaction of maxillary canines. She had class I malocclusion and the suggested treatment plan for her included de-impaction of canine teeth and non-extraction orthodontic treatment. Figure 6 shows the presence of primary canine teeth, and close relationship of the impacted permanent canines with the roots of the central and lateral incisors. The impacted canine teeth were surgically exposed and the brackets were bonded to them. After 1 week, the patient was recalled, and application of distalizing force from the miniscrew placed between second premolar and first molar with chain elastic was started at each side with 150 g force. In the first month, the canine cusp tip emerged through the palatal mucosa. At 3 months, the canine tooth was completely out of the palatal mucosa, and the cantilever spring supported by the mini-screws was placed. Comprehensive orthodontic treatment was started at 6 months. At 11 months, 0.017×0.0 25-inch stainless steel wire were placed in bracket slot and required space for the permanent canines in the dental arch was created with open coil. At 13 months, brackets were bonded to the buccal surface of the canine teeth bilaterally, and they were guided into the dental arch using 0.016-inch and 0.018-inch NiTi overlay wires, respectively. The total duration of treatment was 19 months (Figure 6). 

## Discussion

Treatment of palatally impacted maxillary canines is challenging for clinicians. Schmidt *et al.* [ 27 ] suggested exposure of the palatally impacted canines and allowing them to erupt spontaneously for 6 months prior to the initiation of fixed orthodontic treatment. Natural eruptition of tooth better preserves the surrounding tissue and bone. However, at the end of treatment, if axial inclination of the tooth is not corrected, the results will not be satisfactory. In addition, long course of treatment and inability to control the path of eruption of the tooth are among the disadvantages of this technique.

Becker *et al.* [ 29 ] reported that incorrect detection of the 3D position of the impacted tooth and anchorage loss were the most common reasons for failure of forced eruption of impacted canines. Use of cone-beam computed tomography could greatly help in correct detection of the position of impacted canines in complicated cases [ 14 ]. Some studies have pointed to the benefits of temporary anchorage devices (TADs) for treatment of impacted teeth to reinforce anchorage [ 19 - 20 , 30 - 32 ]. 

We developed a new technique using TADs for treatment of the challenging cases of canine impaction. Our suggested technique has a number of advantages over the conventional technique. Continuous application of appropriate extrusive force by TADs in desired direction, without worrying about anchorage loss, would result in movement of the impacted canine near to dental arch while other tooth is leveled and aligned at the same time, independently. Early application of orthodontic force after the exposure surgery can benefit from the regional acceleratory phenomenon that occurs due to mucosal retraction and bone removal [ 33 ]. Following early application of orthodontic force, duration of treatment significantly decreases and great amount of movement of the impacted tooth will happen at the onset of treatment that is pleasant for patients and motivate them to continue their treatment. Another advantage of our suggested technique is that we can preserve the primary canine teeth for esthetic purposes for a longer period and extract them only when the impacted canines are close to their final position. By doing so, the empty space following the primary canine extraction is soon occupied by the permanent canine, whereas, in the conventional method, buccal mechanics are often used to guide the impacted canine into the dental arch, and thus, the primary canine needs to be extracted at the onset of treatment. 

**Figure 7:**
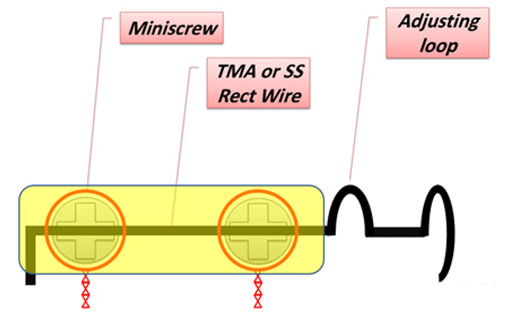
Cantilever spring pervious design

Heravi *et al.* [ 20 ] in their study suggested this novel technique with different appliance design, used two bracket-type mini-screws for treatment of palatally impacted canine teeth. They placed the mini-screws between first premolar and second premolar, and also between second premolar and first molar. A cantilever spring was fabricated using the rectangular TMA wire, passed through the slots of the two bracket-type miniscrews, and ligated with ligature wire. By activating the spring, load was applied to the impacted canine (Figure 7). However, they reported some drawbacks. For instance, they mentioned that surgery and orthodontic force application to the canine tooth in the same session might not be practical in busy orthodontic clinic. In addition, the placement of 2 mini-screws, designing the cantilever spring and wire adapting in the slot of miniscrews is time consuming; while, in case of ankylosis of the canine tooth, it is imperative to apply continuous load as fast as possible after luxation [ 34 ]. Moreover, delay in load application would prevent benefitting from the regional acceleratory phenomenon for acceleration of tooth movement to the maximum [ 33 ]. 

The technique, described in this study, is an improved version of the previous technique with the following advantages. Considering the use of button-type screws, there is no need to pass the cantilever spring through the slots of the bracket-type miniscrews. Thus, placement and adaptation of the cantilever springs would be much easier, and take a little chair time. In addition, in the previous technique, the wire may occasionally disengage from the bracket-type miniscrew slot. This would cause composite fracture, and the spring had to be replaced. However, this can no longer occur in the new technique since the wire is engaged in the hex of the distal screw and is below the contour of the mesial screw. In this new technique, the mini-screws are placed slightly more posterior and between second premolar and first molar, and also first molar and second molar; however, in the previous technique, the mini-screws are placed between teeth first premolar and second premolar, and also second premolar and first molar. Placement of mini-screws between teeth first premolar and second premolar is difficult, and sometimes impossible, due to the inflammation caused by surgical flap elevation; Primary wound healing occurs after 7 days but complete healing can take more. On the other hand, more posterior placement of mini-screws would increase the effective length of the cantilever spring and the mechanical properties of the spring would improve as such [ 32 ]. 

In the this new version of technique, the impacted canine can be easily extruded and distanced from the lateral incisor root by load application from the single mini-screw, and without fabrication of a spring at first. This can be easily performed by a busy clinician. Next, the second mini-screw is added and by employing the cantilever spring, the impacted tooth can be directed to any desired location. 

One of the important advantages of both methods compared to conventional method is the lower risk of root resorption of lateral incisor, regarding the distancing impacted canine from root of incisors before initiating fixed orthodontic treatment [ 19 ]. 

The consent form was also signed by patients or patients’ parents. 

## Conclusion

The suggested new approach for treatment of palatally impacted canine teeth prevents the root resorption of adjacent lateral incisors, and enables evaluation of ankylosis of the impacted canine without placement of fixed orthodontic appliances. Application of TADs shortens the course of treatment and orthodontic force is applied in a controlled manner without any side effect on dental arch form. In this technique, brackets are placed in the oral cavity for a shorter period. In addition, this technique can be easily performed in busy orthodontic offices due to its simple design. It also allows retaining of the primary canine teeth in the oral cavity due to esthetic considerations for a longer period. This technique can be used not only for palatally impacted canines, but also for other impacted teeth in different parts of the oral cavity. 

## Conflict of Interest

The authors declare that they have no conflict of interest.
